# Next-Generation Probiotic Therapy to Protect the Intestines From Injury

**DOI:** 10.3389/fcimb.2022.863949

**Published:** 2022-06-28

**Authors:** Mecklin V. Ragan, Samantha J. Wala, Steven D. Goodman, Michael T. Bailey, Gail E. Besner

**Affiliations:** ^1^ Center for Perinatal Research, Department of Pediatric Surgery, Columbus, OH, United States; ^2^ Nationwide Children’s Hospital, The Ohio State University, Columbus, OH, United States

**Keywords:** *Lactobacillus reuteri*, biofilm, probiotics, necrotizing enterocolitis, *Clostridioides difficile*

## Abstract

Probiotics are live microorganisms that, when administered in adequate amounts, provide health benefits to the host. Some strains of the probiotic *Lactobacillus reuteri* (*L. reuteri*) have both antimicrobial and anti-inflammatory properties that may be exploited for the treatment and prevention of different gastrointestinal diseases, including necrotizing enterocolitis (NEC) and *Clostridioides difficile* (*C. difficile*) infection. Our laboratory has developed a new delivery system for *L. reuteri* in which the probiotic is incubated with biocompatible, semipermeable, porous dextranomer microspheres (DM) that can be loaded with beneficial and diffusible cargo. *L. reuteri* can be induced to form a biofilm by incubating the bacteria on the surface of these microspheres, which enhances the efficacy of the probiotic. Loading the DM with sucrose or maltose induces *L. reuteri* to produce more biofilm, further increasing the efficacy of the probiotic. Using a rat model of NEC, *L. reuteri* administered in its biofilm state significantly increases animal survival, reduces the incidence of NEC, preserves gut barrier function, and decreases intestinal inflammation. In a murine model of *Clostridiodes difficile* infection, *L. reuteri* administered in its biofilm state decreases colitis when administered either before or after *C. difficile* induction, demonstrating both prophylactic and therapeutic efficacy. There are currently no FDA-approved probiotic preparations for human use. An FDA-approved phase I clinical trial of *L. reuteri* in its biofilm state in healthy adults is currently underway. The results of this trial will be used to support a phase 1 clinical trial in neonates, with the goal of utilizing *L. reuteri* in its biofilm state to prevent NEC in premature neonates in the future.

## Introduction

Bacteria grow and adhere to almost every surface, and form complex, multicellular communities called biofilms. Biofilms facilitate successful colonization and maintenance of a bacterial population by protecting bacteria against environmental conditions (Branda et al., 2005). Probiotic bacteria are live microorganisms that can be beneficial to the host when administered in adequate amounts. However, when consumed orally, probiotics face numerous challenges, including the acidic environment of the stomach, effectors of the host immune system, and competition with commensal and pathogenic bacteria. These factors prevent probiotics from being sufficiently sustained within the host, thereby reducing their potential beneficial effects (Navarro et al., 2017). There is extensive, ongoing research examining the use of probiotics for the treatment and prevention of intestinal diseases. In particular, our lab has been investigating the efficacy of the probiotic bacteria *Lactobacillus reuteri* (*L. reuteri*) administered in its biofilm vs. planktonic (free-living) state, to treat necrotizing enterocolitis and *Clostridioides difficile* (*C. difficile*) infections.

*Lactobacillus reuteri* is a Gram-positive bacterium originally isolated in 1962 by German microbiologist Gerhard Reuter. Using human fecal and intestinal samples from infants and adults, Reuter demonstrated that *L. reuteri* was the predominant autochthonous bacterium in both populations. He isolated multiple strains, including DSM20016 (Kandler et al., 1980; Reuter, 2001). In the years since, further genomic studies have demonstrated that human *L. reuteri* strains belong to two distinct multilocus sequence analysis (MLSA) clades, clades II and VI. While human strains in clade VI are more closely related to isolates from chickens, those in clade II are very specific to humans.

Certain rare strains, including DSM20016, are known to have multiple attributes beneficial to the host, including antimicrobial and anti-inflammatory properties (Spinler et al., 2014). The antimicrobial properties of *L. reuteri* are secondary to its ability to produce the antimicrobial compound 3-hydroxyproprionaldehyde (3-HPA), also known as reuterin (Jones and Versalovic, 2009). *L. reuteri* utilizes glycerol dehydratase to produce reuterin from glycerol. Reuterin inhibits the growth of multiple gastrointestinal pathogens, including *C. difficile*, by inducing oxidative stress (Engevik et al., 2020). *L. reuteri* also has extracellular glucosyltransferase (GTF) proteins that catalyze the formation of exopolysaccharides of glucose (glucans) from disaccharide sugars and possess glucan-binding domains that allow for strong binding to other glucans, important to biofilm formation. The anti-inflammatory abilities of *L. reuteri* are partially due to its ability to produce histamine *via* histidine decarboxylase. Histamine is a biologically active compound that modulates host mucosal immunity and suppresses proinflammatory tumor necrosis factor (TNF) production (Jones and Versalovic, 2009; C. M. Thomas et al., 2012). In addition, by metabolizing folate, *L. reuteri* can produce ethionine, which can use ethylation to modify human chromatin (Röth et al., 2019). There is also evidence that *L. reuteri* induces anti-inflammatory T-regulatory cells, suppresses T helper 1 (Th1) and Th2 cytokine responses, and alters dendritic cell activity; however, the mechanisms by which this occurs are poorly understood (Jones and Versalovic, 2009). Our studies use *L. reuteri* strain DSM20016—a strain that possesses both antimicrobial and anti-inflammatory properties.

Biofilms possess a community architecture, replete with a self-made extracellular matrix, and that is often facilitated by adherence to a surface. Biofilm formation enables bacteria to resist environmental conditions, resulting in successful colonization and maintenance of the bacterial population (Salas-Jara et al., 2016). *L. reuteri* grown in its biofilm state on the surface of biocompatible microspheres has an increased ability to survive in acidic environments such as that of the stomach and to adhere to intestinal epithelial cells. The biocompatible microspheres we use are composed of separation pharmacia dextran (Sephadex aka dextranomer microspheres or DM), which are beads of crosslinked dextran typically used for gel-filtration chromatography but also mimic the native glucans that *L. reuteri* makes. In addition, prebiotic nutrients beneficial to the probiotic bacteria can be loaded into the lumen of the Sephadex beads (Navarro et al., 2017). Harnessing the ability of *L. reuteri* to form a biofilm, we have developed a novel probiotic delivery system in which *L. reuteri* is induced to form a biofilm on the surface of DM (*L. reuteri* + DM), allowing for enhanced efficacy. Loading DM with maltose (DM-maltose, which is the substrate for GtfW) or sucrose (DM-sucrose, which is an inducer of *gtfW*) induces increased biofilm formation, enhancing the efficacy of the probiotic (Navarro et al., 2017).

Bacterial colonization of the intestine is essential for the development of a healthy gut microbiome (Harmsen et al., 2000). In contrast to that of term infants, the gut microbiome of premature infants has a notably smaller proportion of beneficial bacteria such as *Lactobacillus* and *Bifidobacteria* and higher numbers of pathogenic bacteria, likely secondary to frequent antibiotic use, exposure to the hospital environment, and artificial feeding. The intestinal dysbiosis that is present in premature infants is associated with the development of necrotizing enterocolitis (NEC) (Mshvildadze et al., 2008).

NEC is a devastating disease affecting premature infants, characterized by extensive intestinal inflammation that often progresses to tissue destruction, bacterial translocation, sepsis, and often death. Approximately 10% of infants born weighing less than 1,500 g will develop the disease, and mortality for affected infants is 20%–30%. Current treatment and preventive approaches for NEC remain suboptimal, with the mainstays of treatment being orogastric tube decompression, total parenteral nutrition (TPN), and administration of broad-spectrum antibiotics. Surgical resection is required when there is a failure of medical management and concern for bowel viability. For infants who survive, they continue to face numerous long-term complications, including gastrointestinal problems, failure to thrive, short gut syndrome, and neurodevelopmental delay (Neu and Walker, 2011).

*C. difficile* infection is also related to a disruption in the gut microbiome. *C. difficile* is one of the most common nosocomial infections. From a 2011 surveillance study in the United States, there were an estimated 453,000 *C. difficile* infections of which 29,300 led to death (Lessa et al., 2015). According to the American College of Gastroenterology, there was a 43% increase in *C. difficile* infection between 2001 and 2012, largely driven by recurrent infections which increased by 188% over the same timeframe (Ma et al., 2017; Kelly et al., 2021). This anaerobic, Gram-positive *Bacillus* is the primary culprit in antibiotic-driven pseudomembranous colitis, which is thought to be due to a disruption of healthy gut microbiota from a single dose or multiple doses of antibiotics. Risk factors that predispose to *C. difficile* infection include hospitalization, age over 65 years, and antibiotic use (Desai et al., 2016; Kelly et al., 2021). Colitis is driven by the release of toxins A and B, both of which are exotoxins that disrupt intestinal cell integrity. Treatment options for patients with active *C. difficile* infection range from oral or intravenous antibiotics, fecal transplantation, and, in fulminant cases of toxic megacolon, emergent total colectomy. Approximately US$5.4 billion is spent annually to treat *C. difficile* infections in the United States (Desai et al., 2016). A prophylactic medication would be extraordinarily cost-effective. However, to date, there is insufficient data to support the use of prophylactic treatments (Kelly et al., 2021).

Given the role of intestinal dysbiosis in the development of NEC and *C. difficile* infections, probiotic administration has been studied as a preventive strategy. However, the multiple studies that have been performed have been met with conflicting results. The Probiotics in Preterm Infants (PiPs) Trial was a multicenter, double-blinded, randomized, placebo-controlled trial conducted from 2010 to 2013 that examined daily dosing of *Bifidobacterium breve BBG-001* in the prevention of NEC, late-onset sepsis, and death in preterm infants while monitoring for probiotic colonization of participants. They found no evidence that the use of *Bifidobacterium breve BBG-001* was protective against NEC, late-onset sepsis, or death in premature infants (Costeloe et al., 2016). However, when used in combination, the probiotics *Lactobacillus* and *Bifidobacterium* spp. have been shown to prevent NEC in very low birth weight (VLBW) infants (Denkel et al., 2016). This is supported by a meta-analysis that showed a reduction in NEC incidence from a combination of *Lactobacillus* and *Bifidobacterium* species among 1,623 VLBW neonates from 8 randomized control trials (Thomas et al., 2017). Another study used the NEO-KISS database, a German surveillance system for nosocomial infections in VLBW infants, to investigate the routine use of a dual-strain probiotic formulation containing *Lactobacillus acidophilus* and *Bifidobacterium* spp. called Infloran in German NICUs from 2004 to 2014. Interestingly, while there is no FDA-approved pharmaceutical-grade probiotic in the United States, Infloran is licensed by the Swiss Agency for Therapeutic Products of the Federal Office of Public Health in Switzerland as a drug for diarrhea, and thus it is available in a pharmaceutical-grade quality. They found that the use of Infloran significantly reduced the risk of NEC, overall mortality, and nosocomial bloodstream infections (Denkel et al., 2016). This is supported by a recent meta-analysis that showed greater NEC reduction with routine multistrain probiotic use compared to routine administration of a single strain (Deshmukh & Patole, 2021). While *Lactobacillus* and *Bifidobacterium* spp. have been shown to prevent NEC in VLBW infants, the positive effects of probiotic administration appear dependent upon repetitive administration (at least daily) (Braga et al., 2011). Although rare, there have been case reports of probiotic-induced sepsis in infants (Zbinden et al., 2015), raising some concern for daily probiotic administration in VLBW infants. A Cochrane review examining 54 studies with a total of 10,604 infants who were either very preterm (born earlier than 8 weeks) or VLBW found a significant reduction in NEC (RR, 0.54; 95% CI, 0.45 to 0.65). However, evidence was assessed as low certainty due to limitations in trial design and publication bias (Sharif et al., 2020).

*Lactobacillus* and *Bifidobacterium* spp. have also been investigated for protective effects against *C. difficile* infection. Although some studies have found beneficial effects, results have been mixed, leading the American Gastroenterology Association to recommend against the use of probiotics for the prevention of antibiotic-induced *C. difficile* (Kelly et al., 2021).

**Figure 1:**
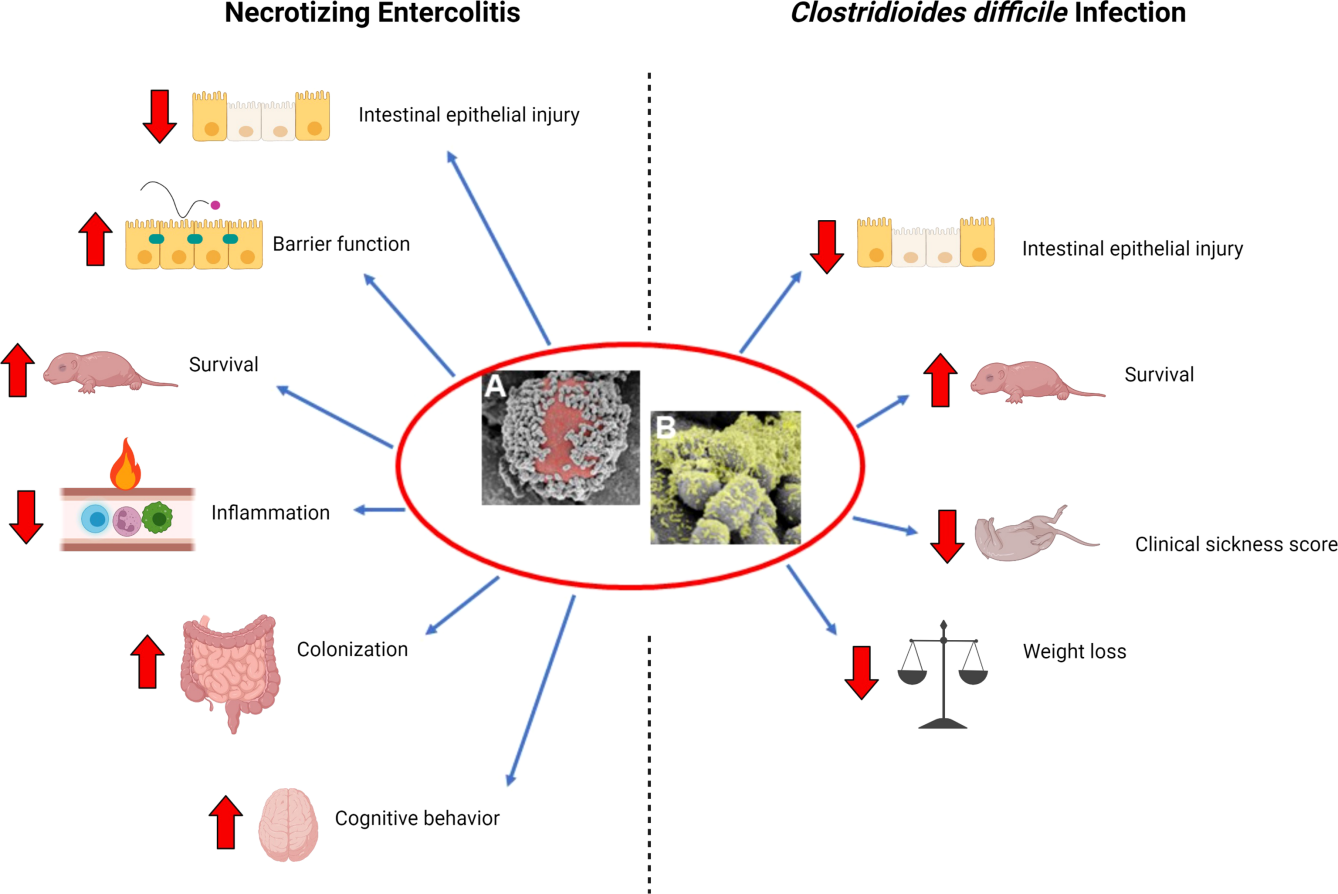
Beneficial effects of *L. reuteri* in its biofilm state. **(A)** Scanning electron microscopy (SEM) image demonstrating multiple *L. reuteri* adherent to the surface of a DM (red); **(B)** upon adherence to DM, *L. reuteri* is induced to form a biofilm (green). Necrotizing Enterocolitis: *L. reuteri* in its biofilm state decreases intestinal epithelial injury, increases gut barrier integrity, increases survival, decreases inflammation, increases probiotic persistence in the gastrointestinal tract, and improves cognitive behavior in a rat model of necrotizing enterocolitis (NEC). *Clostridiodes difficile* infection: *L. reuteri* in its biofilm state decreases intestinal epithelial injury, increases survival, decreases clinical sickness score, and decreases weight loss when administered either as prophylaxis or treatment in a mouse model of *C. difficile* infection. Created with BioRender.com.

Given these concerns and shortcomings of probiotics in human studies, our lab set out to develop an improved method of probiotic administration that would increase probiotic efficacy with fewer doses. Here, we review the currently published literature and progression of our laboratories’ work, which demonstrates the efficacy of a single dose of *L. reuteri* in its biofilm state in the treatment of NEC and *C. difficile* infections. This is not a general review of the field but a focused overview of our laboratories’ contributions to applied probiotic use for these diseases. We also discuss the future implications of this probiotic formulation on the treatment of intestinal diseases (**Figure 1**).

## *L. reuteri* in Its Biofilm State Protects the Intestines From Experimental NEC and Decreases Neurodevelopmental Impairment in Survivors of NEC

Using a rat model of NEC, we have demonstrated that a single dose of *L. reuteri* grown on biocompatible microspheres significantly reduces the incidence and severity of NEC and preserves gut barrier function. In our rat NEC model, premature neonatal rat pups are delivered *via* cesarean section and exposed to repeated episodes of hypercaloric feeding, hypoxia, and hypothermia over a 96-h time period to induce NEC. The hypercaloric diet consisted of 5 daily gavage feedings of increasing volumes of formula each day, with the first feed including a dose of lipopolysaccharide (2 mg/kg) that activates toll-like receptor 4. The first 3 feeds each day were combined with 90-s episodes of hypoxia (<1.5% oxygen) followed by hypothermia (4°C × 10 min). Experimental groups received a single enteral dose of *L. reuteri* grown on unloaded microspheres (biofilm state) compared to *L. reuteri* administered in its planktonic (free-living) state. The intestinal injury was graded using a standardized histology injury scoring system, and intestinal permeability was quantified by measuring serum levels of enterally administered fluorescein isothiocyanate-labeled dextran. Pups treated with a single dose of *L. reuteri* in its biofilm state demonstrated a significant reduction in histologic injury with reduced intestinal permeability, indicating improved gut barrier function, whereas a single dose of planktonic *L. reuteri* had no effect (Olson et al., 2016).

Preloading DM with sucrose or maltose enhances *L. reuteri* biofilm formation, increases *L. reuteri* adherence to human intestinal epithelial cells, and prolongs *L. reuteri* survival in acidic pH (Navarro et al., 2017). With this in mind, we next sought to test the efficacy of *L. reuteri* induced to produce increased biofilm formation by preloading DM with sucrose or maltose, or by mutating the *L. reuteri gtfW* gene to reduce the ability of *L. reuteri* to produce a biofilm, in a rat model of NEC (Olson et al., 2018). GtfW is a strain-specific, cell-associated extracellular enzyme involved in *L. reuteri* biofilm formation (Navarro et al., 2017). Pups in experimental groups received a single enteral dose of *L. reuteri* + DM, *L. reuteri* + DM-sucrose, or *L. reuteri* + DM-maltose. The intestinal histologic injury was graded, intestinal permeability was determined by measuring serum levels of enterally administered fluorescein isothiocyanate-labeled dextran, inflammatory gene expression was determined by quantitative real-time PCR, and preservation of the gut microbiome was determined using DNA isolation and 16S rRNA sequencing. We found that administration of a single dose of *L. reuteri* induced to form increased amounts of biofilm by incubation with either maltose- or sucrose-loaded DM, had significantly improved animal survival, decreased incidence of NEC, reduced intestinal mucosal barrier breakdown, and decreased intestinal inflammation, compared to *L. reuteri* incubated with unloaded DM or *L. reuteri* in its planktonic state. Importantly, *L. reuteri* + DM-maltose led to increased *L. reuteri* persistence in the intestinal tract and caused the composition of the gut microbiome to more closely resemble that of breastfed uninjured animals. Finally, the administration of *L. reuteri* with a mutated *gtfW* gene (i.e., *L. reuteri* is unable to readily produce a biofilm) led to a loss of protection against NEC, highlighting the importance of biofilm formation in the protective effects of *L. reuteri* (Olson et al., 2018).

*L. reuteri* has antimicrobial properties due to its ability to produce reuterin *via* glycerol dehydratase and anti-inflammatory properties partially attributable to its ability to produce histamine *via* histidine decarboxylase. We next investigated the effects of the antimicrobial and anti-inflammatory properties of *L. reuteri* on protecting the intestines from NEC. Prior to the induction of NEC, rat pups received either native or mutant forms of *L. reuteri* in either its planktonic or biofilm states. The mutant forms of *L. reuteri* were either reuterin- or histamine-deficient, decreasing the antimicrobial or anti-inflammatory properties of the probiotic, respectively. The intestinal injury was graded using a standardized histologic injury scoring system (Caplan et al., 1994). As in our prior studies, rat pups that received a single dose of *L. reuteri* in its biofilm state had a significantly decreased incidence of NEC. Administration of reuterin-deficient or histamine-deficient forms of *L. reuteri*, in either the planktonic or biofilm state, resulted in a significant loss of efficacy. This demonstrates the importance of both reuterin and histamine production by *L. reuteri* in its ability to confer intestinal protection against NEC (Shelby et al., 2021). This also highlights both an infectious and an inflammatory etiology of the disease.

There is a growing body of evidence demonstrating a connection between gut microbiota and brain function *via* a bidirectional gut-brain axis, which regulates communication between the enteric nervous system (ENS) and the central nervous system (CNS) (Cryan and Dinan, 2012). Given the known predisposition for infants surviving NEC to have neurodevelopmental delays and cognitive impairments (Neu and Walker, 2011), we next investigated whether our novel probiotic delivery system had neuroprotective effects on survivors of NEC. We induced NEC in rat pups using our standard rat NEC model, as described above. The incidence of death due to NEC in this model is approximately 65%. Surviving pups were placed with foster dams and subjected to daily developmental milestone testing for 23 days. These tests included daily weight, ear and eye opening, auditory startle, tests to measure labyrinthine, body righting, and coordination (air righting, cliff aversion, surface righting, and negative geotaxis), tests to measure strength (forelimb grasp), and tests to measure animal locomotion and the extinguishing of pivoting behavior (open field traversal). In addition, cognitive and memory tests were performed between 4 and 8 weeks of age. These tests included the Y-maze test to assess spatial learning and reference memory, the novel object recognition test to measure nonspatial working memory and recognition memory, the Barnes maze test to evaluate spatial learning and memory, and the elevated plus-maze test to look for anxiety-like behavior. Once collected at the end of the experiment, rat brain specimens were subjected to immunofluorescent staining, RNA isolation, and quantitative real-time PCR. We found that rat pups exposed to NEC reached developmental milestones significantly slower than breastfed pups, with mild improvement when treated with a single dose of *L. reuteri* in its biofilm state. While exposure to NEC was noted to have negative effects on cognitive behavior, these effects were prevented with the administration of a single dose of *L. reuteri* in its biofilm state. In addition, the behavioral effects of NEC were found to be associated with increased numbers of activated microglia, decreased myelin basic protein (MBP), and decreased neurotrophic gene expression. All these effects were prevented by the administration of a single dose of *L. reuteri* in its biofilm state (Wang et al., 2021).

## *L. reuteri* in the Treatment and Prevention of Experimental *Clostridioides difficile* Infection

We have examined the use of *L. reuteri* as prophylaxis and treatment in a murine model of *C. difficile* infection, comparing *L. reuteri* in its biofilm state to its planktonic state (Shelby et al., 2020). To induce *C. difficile* infection, adult C57BL/6 mice received an oral antibiotic cocktail followed by an intraperitoneal injection of clindamycin two days later. Depending on whether the mouse was in the prophylactic or treatment arm of the study, animals received a single dose of saline, planktonic *L. reuteri*, or *L. reuteri* + DM-maltose before or after gastric gavage of 1.5 x 10^7^ CFU of *C. difficile*, respectively, and clinical sickness scores (CSS) and histologic injury scores (HIS) consistent with *C. difficile* colitis were determined (Shelby et al., 2020). In the prophylactic experiment, mice that received a single dose of *L. reuteri* + DM-maltose had significantly decreased weight loss, decreased CSS, decreased HIS, and increased survival after 6 days compared to the control saline group. In addition, mice that received *L. reuteri* + DM-maltose had significantly increased survival after 6 days compared to those that received planktonic *L. reuteri*. In the treatment experiment, animals given *L. reuteri* + DM-maltose had significantly less weight loss, decreased HIS, and increased survival compared to the saline control group. There was a further improvement in CSS and decreased HIS in the *L. reuteri* + DM-maltose group compared to the *L. reuteri* + DM-water group. Therefore, there may be a role for both the prevention and treatment of *C. difficile* infection using *L. reuteri* in its biofilm state (Shelby et al., 2020).

*L. reuteri* has several characteristics that make it effective in the prevention and treatment of *C. difficile* infection. First, it is resistant to the antibiotics used to treat *C. difficile*, such as vancomycin, metronidazole, and fidaxomicin, therefore allowing it to be co-administered with these antibiotics (Spinler et al., 2017). *L. reuteri* is believed to induce reactive oxidative species within *C. difficile* through its production of reuterin, leading to altered metabolism, decreased toxin production, and increased susceptibility to vancomycin and metronidazole (Engevik et al., 2020). Although a randomized controlled trial using planktonic *L. reuteri* in children to prevent diarrhea and antibiotic-associated diarrhea did not show a significant effect when compared to a placebo, it is possible that there would be a clinical difference in the prevention and treatment of *C. difficile* in humans administered *L. reuteri* in its biofilm state (Kołodziej and Szajewska, 2019).

## Human Clinical Trials of *L. reuteri* in Its Biofilm State

We have demonstrated that *L. reuteri* in its biofilm state can reduce the incidence of NEC and *C. difficile* infection in rodent models of disease. However, there are currently no FDA-approved probiotics for human administration despite the increasing use of probiotics in NICUs throughout the United States (Viswanathan et al., 2016). Published clinical trials of probiotics have all used probiotics administered in their planktonic state, with the administration of single or multiple doses daily. Two randomized controlled studies using daily doses of *L. reuteri* DSM17938 found a statistically significant decrease in feeding intolerance and duration of hospitalization for preterm infants weighing ≤1,500 g (Rojas et al., 2012; Oncel et al., 2014). In our animal models, we deliver our formulation of *L. reuteri* in its biofilm state, allowing us to deliver a single dose rather than multiple daily doses. This may help to decrease the risk of downstream bacteremia associated with probiotic administration and increase patient compliance and treatment cost-effectiveness.

In conjunction with Scioto Biosciences Inc., GMP-grade *L. reuteri* in its biofilm state (SB-121) has been produced, and its safety and tolerability are currently being investigated in a randomized, double-blind, crossover phase I clinical trial (NCTT04944901 (*28-Day Daily-Dose Crossover Study of the Safety and Tolerability of SB-121 (Lactobacillus Reuteri With Sephadex^®^ and Maltose) in Subjects, Ages 15 to 45 Years, Diagnosed With Autistic Disorder*, 2021). Autism spectrum disorder is a neurodevelopmental disorder that is characterized by deficits in social behavior and the presence of restricted, stereotyped interests and behaviors. These behaviors involve dysregulation of the gut-brain axis as well as neuroinflammation. In mice subjected to maternal separation, *L. reuteri* DSM 17938 has been shown to downregulate inflammatory gene expression in the brain and enhance pro-social behavior (Park et al., 2021). In addition to neuroinflammation, oxytocin also strongly affects social behavior. Children with autism spectrum disorder have low levels of oxytocin, and in animal models, the administration of *L. reuteri* significantly increases oxytocin and improves social behavior (Kong et al., 2020). These findings support the current phase 1 clinical trial of SB-121 in healthy adults with an autism spectrum disorder. In this trial, volunteers with autism are blindly receiving either daily SB-121 or placebo for 28 days. Those that received SB-121 will then switch to placebo, and those that received placebo will then switch to SB-121 for an additional 28 days. Primary outcome measures will include adverse events, the presence of SB-121 in the stool, and the incidence of symptomatic *L. reuteri* bacteremia. Secondary outcome measures include changes in cognition, attention, and behavior; stool biomarkers; and C-reactive protein and TNF-α levels (since *L. reuteri* can produce anti-inflammatory compounds). The results of this trial should provide reassurance for the safe administration of *L. reuteri* in its biofilm state to not only treat neurodevelopmental disorders like autism spectrum disorder but also to prevent and treat NEC in premature infants and *C. difficile* colitis in susceptible individuals.

## Discussion

*L. reuteri* is a beneficial Gram-positive probiotic bacterium, with strain DSM20016 possessing antimicrobial and anti-inflammatory properties. Importantly, *L. reuteri* can be grown in its biofilm state by using permeable biocompatible microspheres loaded with prebiotics, such as maltose and sucrose, which allows increased adherence to human intestinal epithelial cells and prolonged survival under acidic conditions in the stomach (Navarro et al., 2017). We have demonstrated the efficacy of a single dose of *L. reuteri* administered in its biofilm state in reducing the severity of NEC and *C. difficile* infections in a rat model of NEC and a murine model of *C. difficile*.

While using probiotics to treat intestinal disorders manifested by dysbiosis may seem logical, the reality is more complicated. There are multiple combinations of a variety of probiotics sold over the counter. However, there is currently no FDA-approved probiotic on the market. Still, there is a growing use of probiotics in NICUs in the United States. In 1997, almost no NICUs in the United States were using probiotics (Gray et al., 2020). By 2015, only approximately 14% of NICUs in the United States administered probiotics to VLBW preterm infants (Viswanathan et al., 2016). There are a number of concerns regarding probiotic administration in infants contributing to the difficulty with bringing a product of this nature to market, including probiotic-associated sepsis and contamination, as exemplified by 3 preterm infants who developed *Bifidobacterium longum* bacteremia after receiving the probiotic Infloran^®^ containing viable *Bifidobacterium longum* (Zbinden et al., 2015). The highest profile case involved a preterm VLBW infant who succumbed to gastrointestinal mucormycosis from probiotic ABC Dophilus Powder due to mold contamination with *Rhizopus oryzae* (Vallabhaneni et al., 2015). Beyond concerns for probiotic-associated sepsis, there is also a concern regarding the quality of these probiotic formulations. The precise contents contained within the non-FDA-approved formulations available at present are largely unknown. As recently as 2016, a study was conducted to validate the identity of bifidobacterial species and subspecies in 16 different commercial probiotic products, with only 1 of the 16 probiotics perfectly matching its bifidobacterial label (Lewis et al., 2016). There have also been several recent recalls in the last 5 years of dietary supplement-grade probiotics due to contamination, including with *Salmonella*, *Pseudomonas aeruginosa*, *Rhizopus*, and *Penicillium* species (Liva Global, 2021). These reports highlight the importance of producing a GMP-grade probiotic preparation for human administration. To date, there is no published trial using probiotics to prevent NEC in preterm infants that have met the definition of a “probiotic drug” by the International Scientific Association for Probiotics and Prebiotics (Poindexter and Committee on Fetus and Newborn, 2021). Despite 56 randomized control trials and 30 observational trials, there remains uncertainty about the benefits of probiotics (Razak et al., 2021).

The use of probiotics in the NICU may be far more acceptable to neonatologists if an FDA-approved formulation was available. However, there are concrete reasons why an FDA-approved probiotic product is not currently available for use in the NICU. One of the main challenges is cost. The cost of producing a GMP-grade drug formulation and the effort needed to get it approved by the FDA are enormous. In addition, if the target patient population is newborns, an extra level of scrutiny is appropriately applied, requiring initial phase 1 studies in adults prior to phase 1 studies in newborns, doubling the cost. It would be very challenging to bring a new formulation to the market without the support of biopharma. This brings another level of complexity into the picture. Being an orphan disease, NEC affects a minute portion of the population compared to diseases such as cancer or cardiovascular disease. The impetus for a company to support the development of therapies for NEC is therefore diminished. It is imperative that biopharma understands the importance of cures for orphan diseases in addition to cures for more common diseases affecting humankind.

In our experimental models, a single dose of *L. reuteri* administered in its biofilm state has proven efficacious in reducing the severity of NEC and *C. difficile* infections, and the safety and tolerability of GMP-grade *L. reuteri* in its biofilm state (SB-121) are currently being investigated as part of a phase I clinical trial. Moving forward, further clinical trials will be needed to demonstrate the safety and efficacy of *L. reuteri* in its biofilm state in the prevention and treatment of NEC and *C. difficile* infection.

## Author Contributions

MR and SW have contributed equally to this work and share first authorship. All authors contributed to manuscript revision, read, and approved the submitted version.

## Funding

This work was supported by NIH R01 GM123482 Tunable Native Probiotic Formulations for the Treatment of Necrotizing enterocolitis (GEB, SDG, MTB).

## Conflict of Interest

GB, SG, and MB have stock options in Scioto Biosciences, Inc.

The remaining authors declare that the research was conducted in the absence of any commercial or financial relationships that could be construed as a potential conflict of interest.

## Publisher’s Note

All claims expressed in this article are solely those of the authors and do not necessarily represent those of their affiliated organizations, or those of the publisher, the editors and the reviewers. Any product that may be evaluated in this article, or claim that may be made by its manufacturer, is not guaranteed or endorsed by the publisher.
